# Biomedical applications of iron sulfide-based nanozymes

**DOI:** 10.3389/fchem.2022.1000709

**Published:** 2022-08-29

**Authors:** Yunyi Shan, Wenjie Lu, Juqun Xi, Yayun Qian

**Affiliations:** ^1^ Department of Pharmacology, School of Medicine, Institute of Translational Medicine, Yangzhou University, Yangzhou, China; ^2^ Jiangsu Key Laboratory of Integrated Traditional Chinese and Western Medicine for Prevention and Treatment of Senile Diseases, Yangzhou, China

**Keywords:** nanozymes, iron sulfides, classification, catalytic mechanism, biomedical applications

## Abstract

Nanozymes have attracted great interest owing to their marvelous advantages, such as high stability, facile preparation, and high tunability. In particular, iron sulfide-based nanozymes (termed as ISNs), as one of the most researched nanomaterials with versatile enzyme-mimicking properties, have proved their potential in biomedical applications. In this review, we briefly summarize the classification, catalytic mechanisms of ISNs and then principally introduce ISNs’ biomedical applications in biosensors, tumor therapy, antibacterial therapy, and others, demonstrating that ISNs have promising potential for alleviating human health.

## 1 Introduction

Nanozymes belong to mimic enzymes, which not only possess the unique properties of nanomaterials but also have catalytic activities (Gao and Yan, 2013). Ferromagnetic (Fe_3_O_4_) nanoparticles were first discovered to have the intrinsic peroxidase-like (POD-like) activity, with the similar catalytic process to horseradish peroxidase (HRP) in 2007 (Gao et al., 2007) and then defined in 2013 (Gao and Yan, 2013). Nanozymes have superior properties. For instance, nanozymes are high stability, working in a broader range of pH and temperature. Compared with HRP, Fe_3_O_4_ nanoparticles are certainly stable over a wide range of pH from 1.0 to 12.0 and temperatures from 4.0 to 90.0°C (Gao et al., 2007). Moreover, the preparation of nanozymes is facile, such as hydrothermal synthesis (Xu et al., 2018). Thirdly, nanozymes are conducive to surface modification due to their large surface areas (Miao et al., 2019). Last but not least, nanozymes are high tunability and specific environmental responsiveness (Gao and Yan, 2016; Yang et al., 2021). Based on the advantages described above, frontier research has dramatically promoted the novel applications of nanozymes in sensing, ecological treatment, and biomedicine (Dong et al., 2019; Huang et al., 2019; Sutrisno et al., 2020; Wu et al., 2022; Zhu et al., 2022).

**SCHEME 1:**
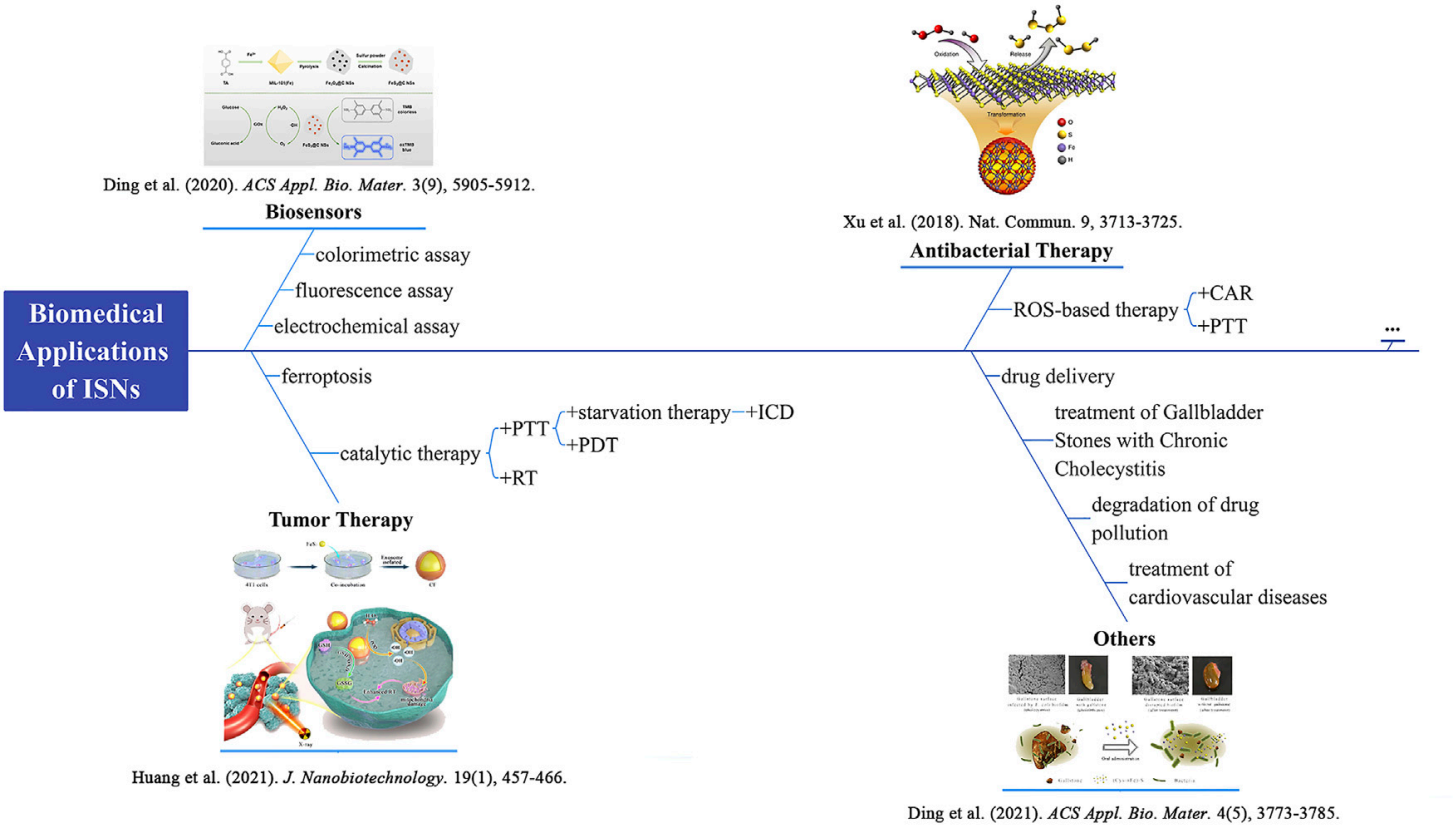
Schematic diagram of biomedical applications of ISNs. Schematics for FeS_2_@C NSs. Reproduced with permission from Ref. (Ding et al., 2020). Copyright ^©^ 2020 American Chemical Society. Schematics for a biomimetic nanozyme system (CF) by coating FeS_2_ into tumor-derived exosomes. Reproduced with permission from Ref. (Huang et al., 2021). Copyright ^©^ 2021 The Author(s). Scheme of polysulfane release from nFeS. Reproduced with permission from Ref. (Xu et al., 2018). Copyright ^©^ 2018 The Author(s). Comparison of cholelithiasis and cholecystitis before and after oral treatment. Reproduced with permission from Ref. (Ding et al., 2021). Copyright ^©^ 2021 American Chemical Society. photothermal therapy, PTT; radiotherapy, RT; immunogenic cell death, ICD; photodynamic therapy, PDT; catalysis-accelerated release, CAR.

With the development of nanozymology, plentiful nanozymes have been discovered or synthesized, such as metal oxides, metal sulfides, and carbon materials. Among these, iron oxide-based nanozymes have been studied and summarized comprehensively, including synthesis, catalytic mechanisms, biomedical applications, and so on (Gao et al., 2017). Nevertheless, iron sulfide-based nanozymes (ISNs) have not been fully studied. What’s more, O and S are congeneric elements. On the one hand, iron sulfides possess similar physiochemical properties as iron oxides; on the other hand, they have their own unique properties, which endow them with special applications in biomedicine. For example, there are more phases of iron sulfides than iron oxides, such as pyrite (FeS_2_), pyrrhotite (Fe_1−x_S), and mackinawite (FeS), while iron oxides have only two forms (Fe_2_O_3_ and Fe_3_O_4_) in nature (Fan et al., 2018; Gao et al., 2019; Kantar et al., 2019; Huang et al., 2021). Notably, ISNs are essential cofactors that serve as active centers for electron transfer in catalytic processes and respiratory chain reactions (Yuan et al., 2020). Therefore, it is predicted that ISNs have huge potential in the biomedical field. Recently, a few studies have focused on the research of ISNs and have made progress in biomedical applications. However, few have thoroughly summarized the intrinsic enzyme-like properties. As a result, it is worthwhile sorting out these study results. In this review, we will try to summarize the classification and catalytic mechanisms of ISNs, and underline their applications in biomedicine (Scheme 1). The work is expected to provide ideas for follow-up research on ISNs.

## 2 Classification of ISNs

The preparation methods of ISNs mainly include hydrothermal synthesis, biomineralization method, co-precipitation, low temperature chemical synthesis, and so on. According to the elemental compositions, ISNs are classified into two categories: 1) different valence states combination of iron and sulfide atoms only, and 2) iron-sulfur compounds doped with other elements.

### 2.1 Different valence states combination of iron and sulfide atoms only

**FIGURE 1:**
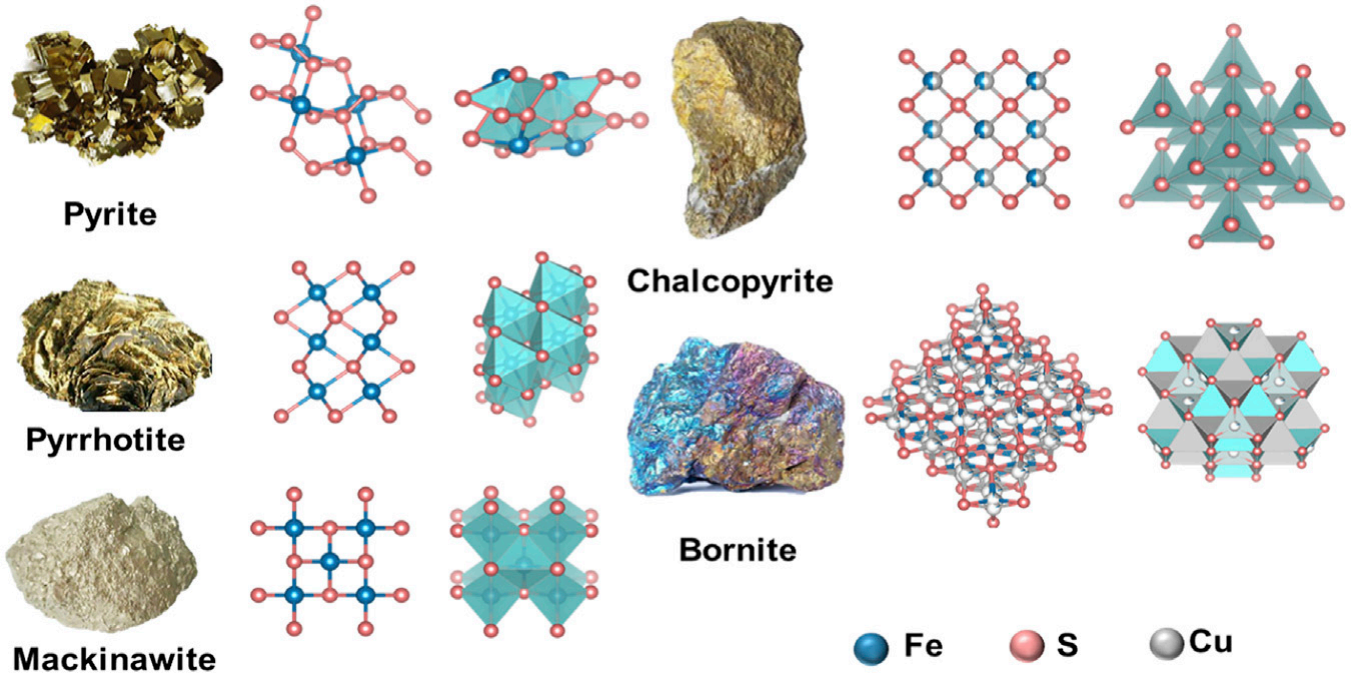
The crystal structure of ISNs from natural minerals. Reproduced with permission from Ref. (Lai et al., 2021). Copyright 2021 Elsevier B.V.

The representative nanozymes in this category are pyrite (FeS_2_), pyrrhotite (Fe_1−x_S), and mackinawite (FeS). Pyrite has a cubic structure with FeS_6_ octahedra and S‒S dimers. The twisted FeS_6_ octahedron has six identical Fe‒S bond distances. A tetrahedral configuration which contains three iron atoms and one S atom is coordinated in each S atom. Pyrrhotite has a hexagonal crystal system, simply described as a twisted octahedral FeS_6_ that forms a three-dimensional (3D) structure. Each FeS_6_ unit shares an edge in the a or c direction with its neighbor. Mackinawite is a kind of tetragonal mineral with a layered structure. The FeS_4_ tetrahedra within the layer connect their adjacent units by sharing corners or edges (Figure 1) (Lai et al., 2021).

### 2.2 Iron-sulfur compounds doped with other elements

Iron-sulfur compounds are usually doped with metallic or (and) non-metallic elements, such as Cu_5_FeS_4_ (bornite), CuFeS_2_ (chalcopyrite), and FeS_2_@C. Cu_5_FeS_4_ and CuFeS_2_ are both doped with a metallic element, which the former crystallizes in the orthorhombic system and the latter has a tetragonal system (Huang et al., 2020; Zhang et al., 2020; Wang X. et al., 2021). FeS_2_@C is doped with the non-metallic element which contains an amorphous nanocomposite (Ding et al., 2020). As far as we know, the crystal form of ISNs affect the catalytic activities. For instance, the FeS_2_ activity of a cubic system is better than CuFeS_2_ that of a tetragonal system (Ltaïef et al., 2018). In addition, in order to improve the activity of nanozymes, researchers tried to modify the surface of ISNs. As proof, Ding et al. designed to wrap two-dimensional (2D) carbon nanosheets on FeS_2_, and the carbon sheets can endow the composites with high specific surface area and abundant active sites to enhance the reaction with substrates (Ding et al., 2020).

## 3 Catalytic mechanisms of ISNs

Up to now, many ISNs have been discovered with distinct enzyme-mimicking properties, including peroxidase (POD), catalase (CAT), oxidase (OXD), and superoxide dismutase (SOD) activities. For ISNs, most of them exhibit POD-like activity (Table 1). Therefore, we emphasize the catalytic mechanism of POD activity. However, the current understanding of the mechanism is still incomplete, and we try to explain it on our limited information. The catalytic mechanism of POD-like activity consists of reactive oxygen species (ROS) generation and the electron transfer process (Yang et al., 2021). On the one hand, ROS include a broad range of chemical species, such as hydroxyl (•OH), hydrogen peroxide (H_2_O_2_), superoxide (•O_2_^−^), and singlet oxygen (^1^O_2_) (Levy et al., 2018). ISNs produce ROS by catalyzing the substrate, which usually trigger the cytotoxicity to fulfill therapeutic effects, such as antibacterial treatment and tumor treatment. For instance, the catalytic mechanism of pyrite (FeS_2_) to produce ROS is divided into the following three pathways: firstly, the active component $\;\equiv \;Fe^{2+}$ react with dissolved O_2_, gradually forming $•O_{2}^{-}$ and releasing Fe^3+^ in aqueous solution (Eq. 1). The resulting Fe^3+^ will be further reduced to Fe^2+^ by $S_{2}^{2-}$ (Eq. 2). Both ≡  Fe^2+^ and Fe^2+^ react with $•O_{2}^{-}$ to form H_2_O_2_ (Eqs 3–4). H_2_O_2_ is finally activated by ≡  Fe^2+^ and ≡ Fe^3+^ (or Fe^2+^/Fe^3+^) to form •OH and hydroxyl peroxide radical (•O_2_H) (Eqs 5–8). Secondly, due to the erosion of H_2_O_2_ on the pyrite surface Fe^3+^ release, which is further reduced to Fe^2+^ by the electron donor S_2_^2−^ (Eq. 9). Then, Fe^2+^ (or ≡  Fe^2+^) and oxidized Fe^3+^ (or ≡  Fe^3+^) by some consecutive reactions with H_2_O_2_ to form •OH and •O_2_H (Eqs 5–8). Thirdly, in the aid of sulfur-defects, the adsorbed H_2_O reacts with surface ≡  Fe^3+^ at a sulfur-deficient site to form the adsorbed •OH, which in turn generates H_2_O_2_ and •OH in aqueous solution. To sum up, the S_2_^2−^ not only promotes the Fe^2+^ dissolution, but also acts as an electron donor to enhance the circulation of Fe^2+^/Fe^3+^ (or ≡  Fe^2+^/ ≡  Fe^3+^). Consequently, pyrite can produce more ROS to apply in antibacterial and antitumor therapies, and so on (Lai et al., 2021). On the other hand, in the electron transfer process, the reduction of H_2_O_2_ or oxygen and the oxidation of substrates are accelerated because ISNs obtain electrons by adsorbing the substrate on the surface and become a wealthy electron donor. This process does not produce •OH or even eliminate its production but can mediately achieve the therapeutic effect (Yang et al., 2021). For example, it is hypothesized that Fe–N–S co-doped porous carbons promoted oxygen reduction by catalyzing the transfer of electrons and protons from organic matter to oxygen. Hence, the reduction product contained $•O_{2}^{-}$ (Borkowski et al., 2020). In addition to POD-like activity, Xu and co-workers proved that nFeS possesses catalase activity which can decompose H_2_O_2_ into oxygen (Xu et al., 2018). Huang et al. discovered a biomimetic nanozyme system in which exosomes from tumors encapsulated by pyrite (FeS_2_) exhibited glutathione oxidase activities which could catalyze glutathione (GSH) into oxidized glutathione (GSSG) (Huang et al., 2021).

$$\equiv Fe^{2+}+O_{2}\to Fe^{3+}+\cdot O_{2}^{-}$$ (1)

$$\mathrm{Fe}S_{2}+14Fe^{3+}+8H_{2}O\to 15Fe^{2+}+2SO_{4}^{2-}+16H^{+}$$ (2)

$$\equiv Fe^{2+}+\cdot O_{2}^{-}+2H^{+}\to Fe^{3+}+H_{2}O_{2}$$ (3)

$$Fe^{2+}+\cdot O_{2}^{-}+2H^{+}\to Fe^{3+}+H_{2}O_{2}$$ (4)

$$\equiv Fe^{2+}+H_{2}O_{2}\to Fe^{3+}+\cdot \mathrm{OH}+OH^{-}$$ (5)

$$\equiv Fe^{3+}+H_{2}O_{2}\to \equiv Fe^{2+}+\cdot O_{2}H+H^{+}$$ (6)

$$Fe^{2+}+H_{2}O_{2}\to Fe^{3+}+\cdot \mathrm{OH}+OH^{-}$$ (7)

$$Fe^{3+}+H_{2}O_{2}\to Fe^{2+}+\cdot O_{2}H+H^{+}$$ (8)

$$2\mathrm{Fe}S_{2}+15H_{2}O_{2}\to 2Fe^{3+}+4SO_{4}^{2-}+2H^{+}+14H_{2}O$$ (9)

**TABLE 1:** Catalytic activities of ISNs. Peroxidase, POD; catalase, CAT; oxidase, OXD; FeS_2_@C nanosheets, FeS_2_@C NSs; FeS@carbon nanosheets, FeS@CNs; glucose oxidase, GOx; paclitaxel, PTX; extracellular matrix-degrading nanoagonist, ECM-dNAc.

Material	Appearances	Catalytic activity	Applications	References
Pyrite (FeS_2_)	Spherical nanoparticles	POD-like activity	Tumor therapy	Kantar et al., 2019; Huang et al., 2021
		and OXD-like activity		
Pyrrhotite (Fe_1-x_S)	Nanosheets	POD-like activity	Pollutant degradation	Gao et al. (2019)
Mackinawite (FeS)	Quasi-spherical nanoparticles	POD-like activity	Antibacterial therapy	Agnihotri et al., 2020
nFeS (Fe_1-x_S, Fe_3_S_4_)	Nanosheets	POD-like activity	Antibacterial therapy	Xu et al. (2018)
		and CAT-like activity		
FeS_2_@C NSs	Nanoparticles	POD-like activity	Biosensers	Ding et al. (2020)
FeS@CNs	Nanoparticles	POD-like activity	Biosensers	Song et al. (2022)
FeS@BSA	Uniform spherical nanoparticles	CAT-like activity	Tumor therapy	Xie et al. (2020)
Chalcopyrite (CuFeS_2_)	Tetragonal	POD-like activity	Pollutant degradation	Huang et al. (2020)
Bornite (Cu_5_FeS_4_)	Orthorhombic	POD-like activity	Pollutant degradation	Zhang et al. (2020)
FeS-GOx@PTX	Uniform nanoparticles	OXD-like activity	Tumor therapy	Ren et al. (2021)
CuS-Fe@polymer nanoparticle	Nanoparticles	POD-like activity	Tumor therapy	Nie et al., 2019
ECM-dNAc	Nanoparticles	POD-like activity	Tumor therapy	Zhan et al., 2022

## 4 Biomedical applications

### 4.1 Biosensors

With the development of biological applications of nanozymes, biosensor has become one of the application branches. Until now, the application of ISNs as biosensors can be divided into three categories: 1) colorimetric assay, 2) fluorescence assay, and 3) electrochemical assay.

#### 4.1.1 Colorimetric assay

The colorimetric assay is mainly based on the principle that ISNs catalyze H_2_O_2_ to form •OH to oxidize substrate 3,3′,5,5′-tetramethylbenzidine (TMB) to blue oxTMB (oxidized TMB), which generates the maximum absorption peak at 652 nm. Therefore, the catalytic activity of ISNs can be utilized to detect the glucose (GLU), glutathione (GSH), cysteine (Cys), and gallic acid (GA) (Song et al., 2022). For example, Ding and co-workers synthesized a hybrid of FeS_2_ nanoparticles encapsulated by 2D carbon sheets (denoted as FeS_2_@C NSs), which was able to detect glucose in more real samples, such as protamine, starch, heparin, lysozyme, and bovine serum albumin (BSA), with a linear range of 0.5–50 µM and detection limit of 0.19 µM (Ding et al., 2020). Song et al. fabricated FeS@CNs, which were capable of detecting H_2_O_2_ content by the colorimetric assay, with a detection limit of 0.78 µM (Song et al., 2022). Accordingly, the FeS@CNs nanozyme-based H_2_O_2_ sensing platform was further constructed to evaluate the antioxidant capacity of GSH, Cys, and GA. Taken together, compared with other colorimetric methods, the colorimetric method using ISNs have the advantages of convenient detection, with the wide linear range and low detection limit.

#### 4.1.2 Fluorescence assay

The fluorescence assay is based on the principle that ISNs effectively catalyze H_2_O_2_ to form •OH to oxidize non-fluorescent substrate to the fluorescent product. For instance, Amplex Red (AR) can be oxidized to oxAR (oxidized AR), which can result in a high fluorescence signal at 585 nm. Therefore, some ISNs can be applied to construct fluorescent biosensors. Song et al. fabricated FeS@CNs, which was also capable of detecting H_2_O_2_ content by detecting the fluorescence signal via the oxAR assay, with the detection limit of 0.86 µM. Notably, antioxidants have the ability of scavenging •OH generated by FeS@CNs, and the presence of antioxidants in the sensing system could reduce the fluorescence signal intensity. Thus, the amount of antioxidants could be quickly evaluated by detecting the fluorescence signals (Song et al., 2022).

#### 4.1.3 Electrochemical assay

**FIGURE 2:**
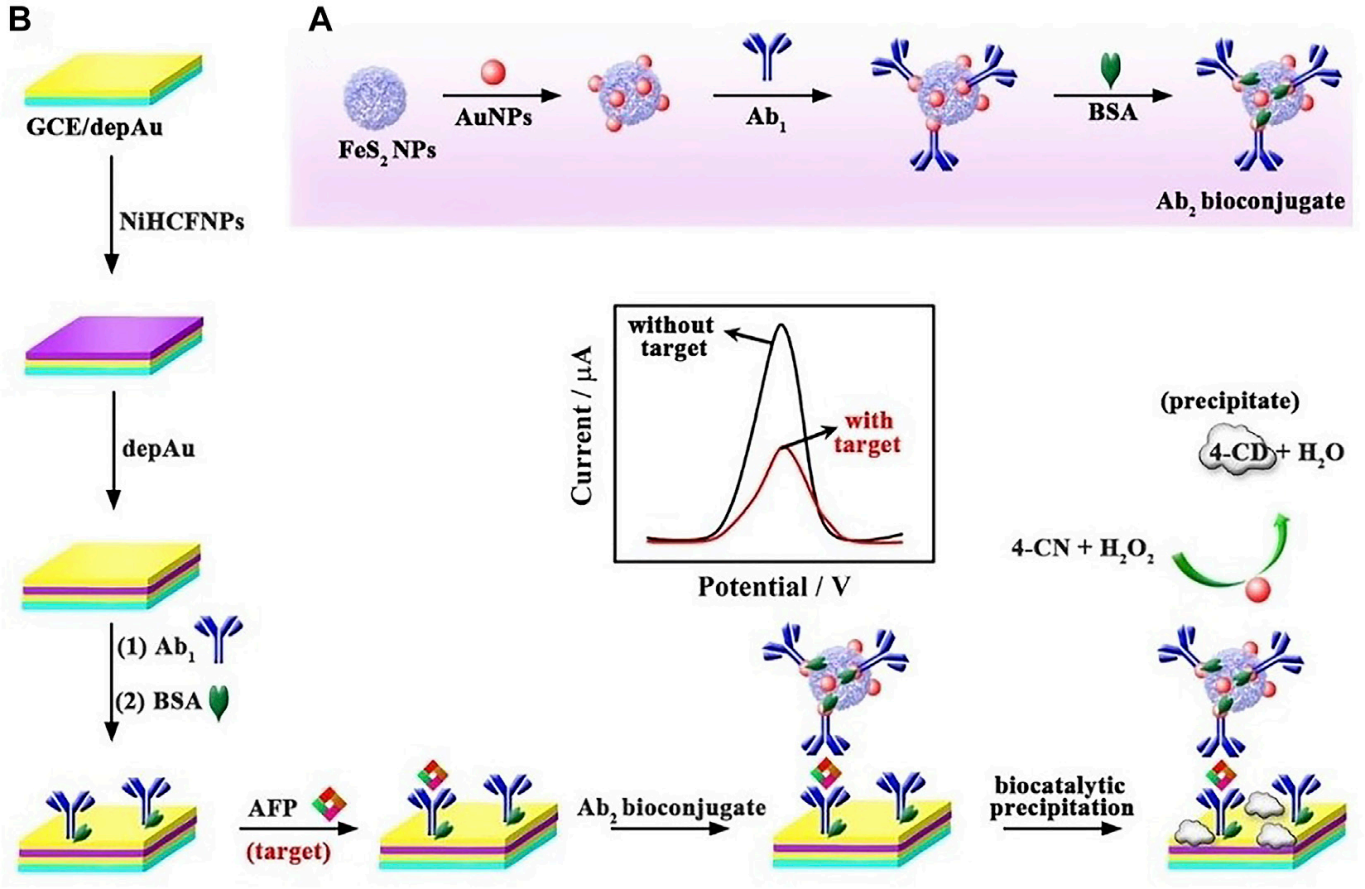
**(A)** Preparation of the FeS_2_-AuNPs-Ab_2_ bioconjugates and **(B)** sandwich model making process for testing AFP. Reproduced with permission from Ref. (Zhang et al., 2019). Copyright ^©^ 2019 Wiley-VCH Verlag GmbH and Co. KGaA, Weinheim.

The electrochemical assay is mainly based on the determination the substance content by measuring electrochemical signals, such as voltage, current, and electricity. Zhang et al. designed a sandwich model including a FeS_2_-AuNPs-Ab_2_ (FeS_2_-Au nanoparticles-antibody) bioconjugate (Figure 2), in which FeS_2_-AuNPs acted as HRP mimicking enzyme to effectively decrease the differential pulse voltammetry (DPV) signal of electroactive materials NiHCFNPs for ultrasensitive detection of *α*-fetoprotein (AFP). Firstly, NiHCFNPs were anchored to the electrode to obtain a strikingly high initial current. Then, FeS_2_-AuNPs composites and Ab_2_ play the role of double hindrance, which greatly reduced the electrochemical signal and improve the ultrasensitive detection of AFP. With the help of this model, AFP in human serum samples could be determined with a linear dynamic and wide range from 0.0001–100 ng/ml. Moreover, the limit detection of this electrochemical assay was 0.028 pg/ml, showing high sensitivity to be used as tumor biomarkers (Zhang et al., 2019).

### 4.2 Tumor therapy

It is well known that tumor-related diseases have become one of the critical diseases that perplex people’s health and lifetime. In recent years, a variety of ISNs for tumor treatments are in full bloom. Presently, the common antitumor therapies based on ISNs are catalytic therapy, ferroptosis, and multiple strategies.

Above all, catalytic therapy refers to iron-based nanoparticles, especially ones containing Fe^2+^, which can catalyze the production of •OH from H_2_O_2_ in tumor cells by Fenton reaction to cause cytotoxicity. For example, Xie and his colleagues showed that FeS@BSA nanoclusters could inhibit hepatocellular carcinoma (Huh7) cells by releasing Fe^2+^ and hydrogen sulfide gas (H_2_S). Specifically, the released Fe^2+^ can produce tumor cytotoxicity through catalytic therapy. Different from iron oxides, the released H_2_S from FeS@BSA nanoclusters also plays a key role in tumor treatment. H_2_S produced by S^2-^ is an endogenous gaseous signal molecule, which plays a vital role in physiological and pathophysiological activities of mammals. Some studies have shown that high concentration of H_2_S can specifically inhibit tumor cells through cellular cycle arrest, miRNA regulation, mitochondrial damage, uncontrolled intracellular acidification stemming from the different metabolic and signal pathways between tumor cells and normal cells. Here, the researchers further discovered that H_2_S effectively inhibited the CAT activity in the Huh7 cells. CAT is a vital antioxidant enzyme in the regulation of intracellular ROS by decomposing H_2_O_2_ to reduce the production of •OH. The failure of cancer treatment is usually related to the high expression of CAT, so inhibiting the expression of intracellular CAT is considered to be a significant means to promote the efficacy of ROS-based catalytic therapy. Here, H_2_S significantly inhibited the CAT activity, which was beneficial for remaining more intracellular H_2_O_2_ for Fe^2+^ to generate •OH, thus enhancing the antitumor effect. This study provided the possibility of gas-amplified tumor therapy achieved by ISN-based therapeutic platform (Xie et al., 2020).

Of course, it is impossible to rely on only one treatment to achieve an excellent antitumor effect. At present, tumor therapy is more advisable when various approaches are used together to play a combined or synergistic effect. In general, various treatments are combined with catalytic therapy. For example, with the combined use of radiotherapy (RT), Huang et al. reported that FeS_2_ wrapped in cancer cell-derived exosomes (CDE) achieved the purpose of synergistic treatment of tumors by catalytic therapy and radiation sensitization (Huang et al., 2021). RT, as is a common method to maintain the life of cancer patients, usually utilize high-energy radiations to cause DNA damage or to accelerate the formation of ROS. Here, FeS_2_ played both glutathione oxidase (GSH-OXD) and POD activities. The former activity reduced the concentration of GSH, an inhibitor of H_2_O_2_ production, to effectively enhance the RT efficacy. The latter activity produced •OH to inhibit the tumor growth. Also, CDE which played a crucial role in targeted tumor therapy has three edges. First, it was unlikely to be cleared by tumor cells because it came from tumor cells. Secondly, it exhibited a high level of non-immunogenicity, so it was barely not swallowed by macrophages. Thirdly, it was easy to penetrate blood vessels to tumor tissue, thus targeting the delivery of drugs (Huang et al., 2021). Above of these, this study showed the potential of ISNs to specifically kill tumor cells. It might provide an idea for the next ISNs targeted therapy of tumor.

Apart from the combination of catalytic therapy and RT, Wu et al. introduced the triple therapy of photothermal therapy (PTT)/starvation therapy/catalytic therapy to enhance catalytic therapy of tumor. They constructed nanocatalysts HPFeS_2_@C (hollow porous carbon coated FeS_2_) composed of tannic acid (TA), glucose oxidase (GOx), and others (Wu et al., 2020). First, TA could reduce Fe^3+^ to Fe^2+^, thus strengthening the Fenton reaction. Secondly, GOx activity of HPFeS_2_@C could promote glucose consumption and produce more H_2_O_2_ to supply the Fenton reaction. At the same time, glucose consumption achieved the goal of starvation therapy. The photothermal effect of the carbon shell which could effectively convert near-infrared (NIR) light into heat could also accelerate the process of Fenton reaction (Wang et al., 2019). In summary, the synergistic triple therapy could greatly improve the effect of catalytic tumor therapy (Wu et al., 2020).

**FIGURE 3:**
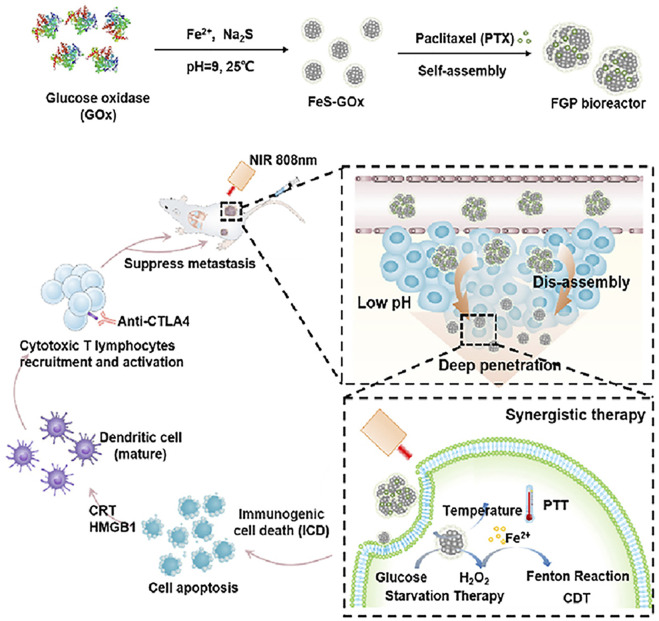
Schematic illustration of the synthetic process of FGP and the mechanism of antineoplastic therapy by applying FGP bioreactor. Reproduced with permission from Ref. (Ren et al., 2021). Copyright 2021 Elsevier.

The study of synergism is not over. Based on catalytic therapy, PTT, and starvation therapy, Ren and coworkers also studied the relationship between ISNs and immunity. The FeS-glucose oxidase@paclitaxel (abbreviated as FGP) gathered in the tumor tissue could broke down into smaller FeS-GOx nanodots with the unique ability to infiltrate into the depths of the tumor tissue due to small size. It was worth mentioning that under the triple action of catalytic therapy, PTT, and starvation therapy dominated by FeS and calreticulin could be exposed to enhance immunogenic cell death (ICD), which has been proved to be a promising method to reverse tumor immunosuppression. Dying tumor cells treated with ICD could release tumor-related antigens and damage-related molecular patterns (DAMPs), which were able to stimulate a specific anti-tumor immune response, promote the maturation of dendritic cells (DCs) and recruit special T cells, and ultimately inhibit metastasis of tumor cells with the cooperation of anti-cytotoxic T-lymphocyte-associated protein 4 (anti-CTLA4) checkpoint blockade. This revealed that their work could be used as an effective treatment for inhibiting the metastasis and recurrence of tumor (Figure 3) (Ren et al., 2021).

Additionally, photodynamic therapy (PDT) is another strategy of tumor treatments. Feng et al. designed a FeS_2_@sorafenib@bovine serum albumin (FeS_2_@SRF@BSA) nanoplatform that combined catalytic therapy, PTT and PDT to realize “all-in-one” nano reagents. Among them, the role of catalytic therapy is no longer discussed, focusing on the therapeutic effect of PDT. Photodynamic reaction of FeS_2_@SRF@BSA under 808 nm laser produced ^1^O_2_ that was also a kind of ROS. Simultaneously, PTT also occurred at the same wavelength and then loaded with chemotherapeutic drugs SRF, so it finally cooperated to inhibit tumor growth (Feng et al., 2022).

**FIGURE 4:**
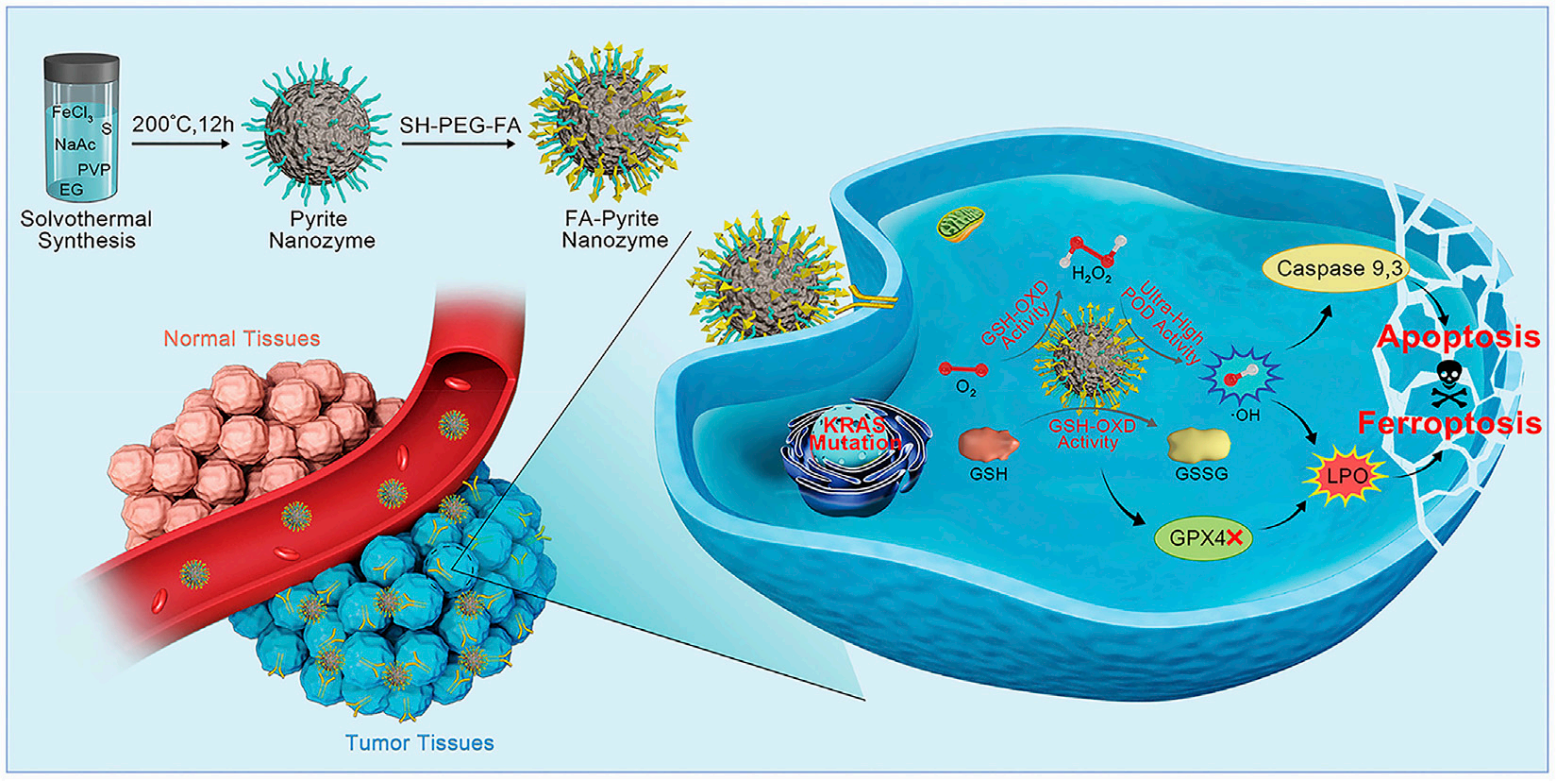
Schematic illustration of apoptosis−ferroptosis synergistic tumor therapy by using pyrite nanozymes with ultrahigh POD-like catalytic activity and intrinsic GSH-OXD mimicking ability. Reproduced with permission from Ref. (Meng et al., 2021). Copyright ^©^ 2021 American Chemical Society.

Compared with catalytic therapy, ferroptosis is a relatively new concept, which refers to one of the regulated forms of cell death. Lipid hydroperoxidase glutathione peroxidase 4 (GPX4) plays a cardinal role in ferroptosis, which can use cofactor GSH to reduce the reactive lipid hydroperoxides (LPO) to inactive lipid alcohols, thus avoiding the production of toxic lipid ROS which lead to membrane damage and ferroptosis. Therefore, the inhibition of GPX4 by reducing GSH has become a novel idea of tumor therapy (Yang and Stockwell, 2016; Meng et al., 2021). Meng, Li and colleagues reported that pyrite (FeS_2_) exhibited POD-like activity and GSH-OXD-like activity (Figure 4). The former activity produced •OH for apoptosis, while the latter activity triggered ferroptosis of tumor cells, which bypassed the inhibition of apoptosis that often occurred in many tumor cells. Remarkably, they creatively summed up the features of FeS_2_ in the treatment of tumors. First, FeS_2_ exhibits extremely high affinity to H_2_O_2_, and its POD catalytic efficiency is 4,144- and 3,086-fold higher than that of classical Fe_3_O_4_ nanozyme and HRP, respectively. This makes it possible to make full use of the limited H_2_O_2_ in the tumor microenvironment to produce sufficient •OH for tumor therapy. Second, glutathione oxidase (GSH-OXD)-like activity was found in FeS_2_, which could oxidize GSH to produce H_2_O_2_ for catalytic therapy. Thus, a self-cascade platform to produce plentiful •OH is realized. Lastly, since tumor cells have more active metabolism and produce more H_2_O_2_ and have higher content of GSH and iron dependence, FeS_2_ gives priority to killing tumor cells, which means that it has tumor-specific cytotoxicity (Meng et al., 2021). Similar antitumor treatments include biogenic FeS NPs, as well as a biomimetic nanozyme system, in which exosomes from tumors encapsulated by pyrite (FeS_2_) (Dang et al., 2021; Huang et al., 2021).

Furthermore, the surface modification of ISNs is necessary because the bare nanoparticles are small and easy to be eliminated by the kidney (Wang Z. et al., 2021). Usually, macromolecules, such as polyethylene glycol (PEG), are adopted to increase ISNs’ volume and prolong the time which it takes to function in the body. Some small molecules could also be used for the surface modification of ISNs. For example, paclitaxel, as a chemotherapeutic drug, was be able to co-assembled into FeS-GOx through hydrophobic interaction, which not only played a synergistic effect, but also prolonged the residence time of FeS-GOx in the tumor site (Ren et al., 2021). Additionally, in order to enhance the specificity of ISNs, researchers have tried to add targeted substances to the surface, such as folic acid (FA), tumor cell secretions and so on. For instance, the above-mentioned CDE is an endogenous vesicle with a size of 50–200 nm, and its three advantages make the material achieve considerable results in targeted tumor therapy (Huang et al., 2021). However, in some cases, surface modification may result in adverse effects. For example, the function of the FA-pyrite nanozymes modified by PEG was affected (Meng et al., 2021).

### 4.3 Antibacterial therapy

With the abuse of antibiotics and the production of superbugs, many existing antibiotics have a poor bactericidal or bacteriostatic effect, so there is an urgent need for more effective antimicrobials to be put on the market. In antibacterial treatments, some ISNs have achieved certain curative effects. For example, pyrrhotite (Fe_x-1_S) nanoplates were synthesized, and it was found that ROS in bacteria enhanced when Fe_x-1_S nanoplates were exposed to H_2_O_2_ and air. It showed germicidal performance against *Escherichia coli*, *Staphylococcus aureus*, and *Enterococcus faecalis*, which confirmed that Fe_x-1_S nanoplates exhibited a good bactericidal effect (Argueta-Figueroa et al., 2018).

With the further development of the research, ISNs not only regulate the level of H_2_O_2_ in the organism to affect the survival of bacteria but also kill bacteria through catalysis-accelerated release (CAR), as discovered and named by Xu et al. CAR refers to the rapid oxidation of the surface of nFeS (Fe_1-x_S, Fe_3_S_4_) in the presence of H_2_O_2_, accelerating the release of polysulfanes. Polysulfanes have been proved to have the bactericidal ability, so the nFeS has the ability to kill a variety of pathogenic drug-resistant bacteria, such as Gram-negative bacteria (*Pseudomonas aeruginosa, Escherichia coli*), Gram-positive bacteria (*Staphylococcus aureus*) and drug-resistant strains of *Staphylococcus aureus*, which could be used in the treatment of biofilm on human teeth and promote wound healing. Moreover, the researchers illustrated that the release of polysulfanes might be the general feature of ISNs antibacterial therapy, which provided a new idea for ISNs in treatment of bacteria and even the whole biomedical applications (Xu et al., 2018; Liu and Qu, 2019).

In addition, some researchers introduced infrared laser based on the production of ROS through dual-modality therapy to achieve a better antibacterial effect. It is worth noting that although FeS is insoluble in water. FeS nanoparticles (200 μg/ml) synthesized in the aqueous phase, for example, can permanently release Fe^2+^ in an aqueous dispersion. Specifically, with the help of the ferrozine assay, the release curve of Fe^2+^ shows a time-dependent manner of rapid release at first and then stable release. Combined with visible and NIR light exposure, it caused significant hyperthermia and showed that the level of intracellular ROS was gradually increasing (Agnihotri et al., 2020).

Moreover, the carbon nanospheres (CNSs) with the decoration of ultrasmall FeS_2_ nanoparticles (denoted as CNSs@FeS_2_) reported by Xi and partners also used similar antibacterial mechanisms. Notably, they pointed out that the role of sulfur was to protect Fe^2+^ and ensure the antibacterial effect of Fe^2+^. Furthermore, it was also proved that different valences sulfur ions would determine whether they have a direct bactericidal activity or not. For instance, S_2_^2−^ and S^2−^ had no direct germicidal ability. However, it was found that other polysulfides, such as S_3_^2−^ and S_4_^2−^ exhibited excellent bactericidal activity (Xi et al., 2021).

### 4.4 Others

Apart from biosensors, tumor therapy, and antibacterial, ISNs are also applied in other biomedical fields, such as cardiovascular diseases therapy, drug degradation, treatment of some digestive diseases, drug delivery systems, and so on.

About treatment of cardiovascular diseases, it has been proposed that the utilization of PTT is able to treat artery inflammation and stenosis using AgFeS_2_ nanoparticles. AgFeS_2_ nanomaterial can take therapeutic effects at very low concentration and have been demonstrated to be safe for cells and animals (Peng et al., 2020). With regard to the degradation of drug pollution, the photocatalyst FeS_2_-Bi_2_O_3_ was proved to have a high degradation rate of 97.5% for phenytoin sodium under 50 min of ultraviolet light. It is worth noting that FeS_2_-Bi_2_O_3_, as a photocatalyst, is only reduced by about 3% after five rounds of reuse, which significantly enhances the performance-to-price ratio (Pasha et al., 2019). Additionally, with the economic level gaining steam, humans’ quality of life has also improved, a double-edged sword. On the one hand, people’s happiness index has enhanced dramatically. On the other hand, obesity, hyperlipidemia, aging population are also coming. An increasing number of people suffering from gallstones every year stems from these factors (Yao et al., 2021). To treat cholelithiasis and cholecystitis, compared with traditional clinical drug therapy, Ding and his co-workers found that oral nFeS supernatant cleared gallstones and reduced the bacteria in the gallbladder by 60% after 2 days of treatment in mice. In addition to the remarkable treatment result, nFeS supernatant showed high biocompatibility and had the potential to be used as a nano-preparation for the treatment of cholelithiasis and cholecystitis (Ding et al., 2021). In terms of drug delivery, studies have shown that ferrihydrite and concentrated sulfuric acid undergo unusual chemical reactions to form hematite nanoparticles, which can be targeted at red blood cells, which means that if the nanoparticles are loaded with drugs, then they are able to be transported to various parts with the red blood cells. In addition, it can also target tumor cells. Ultimately, its application can be extended to nano-sensing, imaging, therapy, and so on (Lunin et al., 2020).

## 5 Conclusion

To sum up, we have briefly summarized the research progress of ISNs in recent years from three aspects: classification, catalytic mechanisms, and biomedical applications. Due to their simple preparation, excellent catalytic activities, and high tunability, ISNs have a broad development prospect, such as biosensors with higher sensitivity, biological agents with better inhibition of tumor growth, and new antibiotics with better bactericidal efficiency. Remarkably, the existence of sulfur element in ISNs make it different from other iron-based nanozymes and has its own unique biomedical applications. Combined with the current research results, sulfur has the following three major functions: 1) Sulfur element facilitates the circulation of Fe so that ROS can be formed persistently; 2) Sulfur element, as a donor of H_2_S, participates in the treatment of tumors; 3) As the source of polysulfanes formation, sulfur element helps to improve the killing ability of bacteria. Nevertheless, it is undeniable that there are still some problems to be solved in ISNs. First of all, ISNs, generally speaking, are not very toxic and has little effect on normal physiological activities while exerting curative effect in animals. However, the toxicity estimation of ISNs needs to be studied systematically. The metabolic pathways and degradation process should be studied in detail to assist in the assessment of ISNs’ toxicity. Theoretically, ISNs can be encapsulated by lipid carriers or modified by other biomolecules (such as PEG) to fabricate drug delivery systems to improve the dispersity and reduce the toxicity. Nevertheless, the effects of lipids or PEG on catalytic activities of ISNs should also be investigated carefully. Secondly, the specificity of ISNs which only kills tumor cells and has little effect on normal cells is expected to come true. If this point is resolved, the damage to human health caused by indistinguishable attacks on tumors and normal tissues by conventional treatments such as chemotherapy can be addressed. However, the half-life of ISNs in tumor targeted therapy is relatively short due to their fast degradation in serum, renal clearance and liver metabolism. So, the therapeutic effect is not satisfactory. How to extend the therapeutic half-life of ISNs is well worth considering. Furthermore, the role of sulfur in ISNs needs to be further explained by more studies. Moreover, the current research shows that ISNs have an excellent application prospect in tumor therapy, antibacterial, and other major diseases troubling human health, so whether they can really be applied in clinical treatment still need to spend an abundance of time. Last but not least, other biomedical applications of ISNs are worth digging into. These problems need more researchers to devote themselves to more scientific research. We are looking forward to the biomedical applications of ISNs to cure diseases and benefit humanity in the near future effectively.
